# *Shewanella* spp. infections in Gran Canaria, Spain: retrospective analysis of 31 cases and a literature review

**DOI:** 10.1099/jmmcr.0.005131

**Published:** 2017-12-07

**Authors:** Alberto J. Martín-Rodríguez, Oriol Martín-Pujol, Fernando Artiles-Campelo, Margarita Bolaños-Rivero, Ute Römling

**Affiliations:** ^1^​ Department of Microbiology, Tumor and Cell Biology, Karolinska Institutet, Stockholm, Sweden; ^2^​ Instituto Universitario de Biorgánica “Antonio González”, Universidad de La Laguna, Tenerife, Spain; ^3^​ Hospital Universitario Insular de Gran Canaria, Gran Canaria, Spain; ^4^​ Hospital Universitario de Gran Canaria ‘Doctor Negrín’, Gran Canaria, Spain; ^†^​ Present address: Swedish Medical Nanoscience Center, Department of Neuroscience, Karolinska Institutet, Stockholm, Sweden.

**Keywords:** *Shewanella*, nosocomial, soft-tissue infection, bacteremia, patera foot, multidrug resistant

## Abstract

**Introduction:**

*Shewanella* spp. can cause severe skin and soft-tissue infections, gastrointestinal infections, otitis and bacteraemia, generally upon contact with seawater or consumption of raw seafood. Recently, a new condition termed ‘patera foot’ characterized by acute skin and soft-tissue infection has been described in irregular immigrants arriving to the Canary Islands, Spain, in rudimentary boats. Most infections are caused by a single species, *Shewanella algae*. The improvement of the diagnostic capabilities in clinical microbiology laboratories has resulted in a growing number of cases being reported worldwide, most of them coming from warm regions.

**Case presentation:**

In this work, we reviewed the medical records of all the patients with *Shewanella* infections in the two university hospitals of Gran Canaria (the Canary Islands, Spain) during the period 2000–2016, resulting in the identification of 31 cases. We also conducted a literature review of *Shewanella* infections reported worldwide in recent years.

**Conclusion:**

This case series suggests that *Shewanella* infections are nosocomially acquired more frequently than previously thought. In addition, the unexpectedly high proportion of multidrug-resistant isolates raises concerns.

## Introduction

*Shewanella* spp. are Gram-negative rods abundant in marine and freshwater environments [1]. While the broad respiratory versatility of this genus has been intensively researched for biotechnological and environmental applications [2], only a few *Shewanella* species have been described as causative agents of human infections. Previously, *Shewanella* spp. had often been misidentified in the clinical microbiology laboratory, as API test strips were not sufficiently discriminatory for clinically relevant *Shewanella* spp. [3], leading to *Shewanella algae* being misidentified as *Shewanella putrefaciens*. Refined identification procedures have diagnosed a growing number of cases that required re-evaluation of the pathogenic potential of certain *Shewanella* spp., in particular *S. algae*. Current state-of-the-art knowledge indicates that one species, *S. algae*, is responsible for around 80 % of all cases [3]. The molecular mechanisms enabling *S. algae* to cause infections are unknown. *S. algae* is often isolated in the context of polymicrobial infections; thus, its clinical impact has not been fully defined. However, *Shewanella* infections are still largely considered exceptional.

Herein, we report the experience from Gran Canaria, the Canary Islands, Spain. We retrospectively reviewed all cases of *Shewanella* infections diagnosed during the period 2000–2016 at the two university hospitals, Hospital Insular and the Hospital Doctor Negrín, that serve the island of Gran Canaria, with a population of approximately 850 000. We have covered the epidemiology, microbiology and outcome of the infectious episodes, and discuss our findings from a microbial ecology perspective. In addition, we conducted a literature update on recently reported cases of *Shewanella* infections worldwide.

## Methods

### Patients

We conducted a retrospective analysis of the medical records from 2000 to 2016 at the Hospital Insular and Hospital Doctor Negrín, Las Palmas de Gran Canaria, Spain. Demographic and epidemiological data from the patients were retrieved. Microbiological data included species identification, co-isolates and antimicrobial susceptibility, as well as clinical data and the outcome of the infection episode.

### Microbiology

Bacterial identification and antibiotic-susceptibility testing differed between the two hospitals and over time, and was conducted as stated below.

#### Hospital Universitario Insular de Gran Canaria

Since March 2014, *Shewanella* spp. have been identified by matrix-associated laser desorption ionization-time of flight (MALDI-TOF) MS. Before this time, all *Shewanella* isolates were initially identified as *S. putrefaciens* by using the API20E/API20NE system (bioMérieux). At all times, phenotypic characteristics such as growth at 42 °C, growth in 6.5 % NaCl and β-haemolysis were identification criteria for *S. algae*. MALDI-TOF MS identification results were further validated using the Vitek-2 automatic system, as described below.

*In vitro* susceptibility testing was performed using the disc-diffusion method on Mueller–Hinton agar with an inoculum corresponding to a McFarland 0.5 turbidity standard. Readouts were taken after 18 h of incubation at 35 °C. Isolates were considered resistant (R), intermediate resistant (I) or susceptible (S) to the tested antibiotics following the interpretative standards of the Clinical and Laboratory Standards Institute (CLSI) for 'other non-Enterobacteriaceae' [4].

#### Hospital Universitario de Gran Canaria ‘Doctor Negrín’

Bacterial identification and antibiotic-susceptibility testing of the isolates was performed with the Vitek-2 (bioMérieux) automatic system. Species identification was considered positive if the type index was 0.95 or higher. The isolates were ranked resistant (R), intermediate resistant (I) or sensitive (S) to the tested antibiotics as described in the CLSI guidelines [4].

## Results

### Case series in Gran Canaria, Spain

A total of 31 cases occurred in 31 patients with the main clinical manifestations summarized in Table 1. Twenty-three patients (74.2 %) were men and eight (25.8 %) women. The mean age of the patients was 50.7 years, ranging from 15 to 87 years. Isolates were from skin and soft-tissue infections (18), blood (8), peritoneal lesions (2), bronchial aspirate (1), bile (1) and ear swab (1). A total of 15 of the 31 isolates had been identified as *S. putrefaciens*; the other 16 were identified as *S. algae*. Fourteen of the thirty-one isolates were from polymicrobial infections with other opportunistic Gram-positive and Gram-negative pathogens. Nine infections were associated with irregular African refugees arriving in rudimentary boats after a transoceanic journey lasting several days in overcrowded conditions. During this time, they had been exposed to weather inclemencies, water splashes and prolonged immersion of their feet in seawater coming into the boats, possibly contaminated with traces of urine, faeces and dirt. Otherwise, recent exposure to seawater was only unambiguously associated with one additional case, and could not be retrieved from the history of 20 patients. One wound infection case was associated with sand contamination from a local park.

**Table 1.** Characteristics of *Shewanella* spp. infections in Gran Canaria between 2000 and 2016

Case	Date	Origin	Isolate	Co-isolate	Age (years)	Sex	Exposure to seawater	Sample	Underlying condition	Clinical findings	Treatment	Outcome
1	27/10/00	C	*S. putrefaciens*	N	57	M	NS	Ear swab	AHT, DM	Severe external otitis	NS	Favourable
2	19/02/01	C	*S. putrefaciens*	N	68	M	NS	Blood	AHT, DM, dyslipidaemia, IC (bypass)	Fever while hospitalized at Neurology department	NS	Favourable
3	27/05/03	NSC	*S. putrefaciens*	*E. coli*	70	M	NS	SST (abscess)	Colon carcinoma, AHT, DM, IC (bypass surgery)	Rectal bleeding, diarrhoea, nausea, epigastric pain, surgical wound infection	NS	NS
4	01/12/03	NSC	*S. putrefaciens*	N	56	F	NS	Blood	AHT, IC, DM, vascular disease (2 stents)	Fever while hospitalized at Cardiology department	Gentamicin+ceftriaxone	Favourable
5	06/03/04	C	*S. putrefaciens*	*Acinetobacter haemolyticus*	32	F	NS	Blood	Severe asthma, hiatal hernia, gastroesophageal reflux, obesity, hypercholesterolaemia	Fever while hospitalized	Levofloxacin	Favourable
6	23/03/04	C	*S. putrefaciens*	N	64	M	NS	Blood	Lung cancer (lobectomy, 2001), DM, asthma	Pericardial effusion and pericarditis, hospitalized at Cardiology department	NS	Favourable
7	10/02/05	C	*S. putrefaciens*	N	80	F	NS	Blood	Colon and cervix carcinoma, cholecystectomy, pancreatic mass of unknown origin	Nausea, vomiting, fever, abdominal pain; hospitalized at Oncology department	Antibiotics (NS)	Favourable
8	28/08/06	NSC	*S. putrefaciens*	N	67	F	NS	Blood	DM, chronic renal failure (renal transplant), AHT, IC	Sepsis that required intensive care	Antibiotics (NS)	Death (unrelated to infection)
9	29/06/07	C	*S. putrefaciens*	*Staphylococcus aureus*	20	M	**Y (patera)**	SST (abscess)	Septic shock, renal failure, rhabdomyolysis	Hypothermia after patera sinking; septic shock, cellulitis in inferior limbs; malodorous purulent ulcer in a finger	Antibiotics (NS)	Surgical debridement of the lesions; amputation of the finger; otherwise favourable
10	21/08/07	C	*S. putrefaciens*	N	25	M	**Y (patera)**	SST (cutaneous wound swab)	–	Necrosis of the left foot (dorsal); multiple wounds on the left thigh	Piperacillin/tazobactam+doxycycline, followed by cefazolin+cephalexin	Surgical debridement of the lesions; amputation of several fingers and skin graft; slow recovery, otherwise favourable
11	13/08/08	C	*S. putrefaciens*	N	74	M	NS	Blood	AHT, colon carcinoma, liver and lung metastases, cholecystectomy	Sepsis of abdominal origin; high fever, vomiting, diarrhoea, unconsciousness, dehydration	Piperacillin/tazobactam+levofloxacin	Favourable
12	04/09/08	C	*S. algae*	N	21	M	**Y (patera)**	SST (ulcer)	Septic shock, renal failure, rhabdomyolysis	Multiple dermal erosions in the legs	Linezolid	Favourable
13	05/09/08	C	*S. algae*	*Bacteroides thetaiotaomicron*	24	M	**Y (patera)**	SST (phlyctena)	–	Dehydration, rhabdomyolysis, contusions in the legs	Ceftazidime+doxycycline	Debridement, skin graft, amputation of one phalanx and two fingers; slow recovery, favourable
14	09/09/08	C	*S. algae*	*A. salmonicida*	20	M	**Y (patera)**	SST (cutaneous biopsy)	–	Vomiting and diarrhoea after ingestion of seawater; anorexia (6 days), fever, dehydration, cutaneous excoriations, tumefaction, cellulitis in left leg with phlyctenas	Imipenem+ceftazidime+ doxycycline+clindamycin	Debridements, amputation of several fingers; slow recovery, favourable
15	15/09/08	C	*S. putrefaciens*	N	20	M	**Y (patera)**	SST (wound)	General health deterioration	Right knee arthritis	Cefotaxime+amoxicillin/clavulanic acid	Favourable
16	09/12/08	C	*S. algae*	*Staphylococcus aureus*, *Morganella morganii*	20	M	**Y (patera)**	SST (wound)	–	Pain and tumefaction in the right arm and leg; necrotic phalanx (4th finger, right hand)	Antibiotics (NS)	Amputation of the necrotic finger; otherwise favourable
17	20/08/10	C	*S. algae*	N	72	M	NS	SST (exudate from catheter site)	Chronic renal disease, renal transplant (1999), peritoneal dialysis catheter (2010), multiple myeloma, osteoporosis	Exudate around the dialysis catheter insertion area; slight infection	NS	Favourable
18	04/09/10	C	*S. putrefaciens*	N	70	M	NS	Blood	AHT, auricular fibrillation, myocardial infarction (1996), 2 stents	Fever	NS	Favourable
19	14/03/11	C	*S. algae*	N	70	M	NS*	SST (abscess)	AHT, dyslipidaemia, chronic liver disease, alcoholism	Traumatic wound in the left parieto-occipital region with necrotic, malodorous areas; fever	Ciprofloxacin+vancomycin+ amoxicillin/clavulanic acid	Favourable
20	05/10/11	NSC	*S. algae*	N	87	M	NS	Bronchial aspirate	DM, Parkinson’s disease, AHT, peritonitis with colostomy, bacteraemia (*Pseudomonas aeruginosa* XDR, 2011)	Complicated acute cholecystitis; accumulation of secretions in both lungs	NS	NS
21	06/06/12	C	*S. algae*	N	50	M	NS	SST (gluteal abscess)	Crohn’s disease with ileostomy and proctocolectomy, intermittent jaundice, hypertriglyceridaemia, DVT; treatment with infliximab since 2009	Gluteal abscess after a fall; perineal sinus	NS	Torpid but favourable
22	23/05/13	NSC	*S. algae*	*K. oxytoca*, *E. coli*, *Bacteroides fragilis*	73	F	NS	Peritoneal lesion	AHT, biliary fistula, adenocarcinoma of the ampulla of Vater	Abdominal drain infection; gastrostomy	Metronidazole+other antibiotics (NS)	Favourable
23	02/06/14	C	*S. putrefaciens*	*K. oxytoca*	40	F	NS	Peritoneal lesion	Chronic renal failure	Acute peritonitis in patient with peritoneal dialysis; fever	NS	Favourable
24	07/08/14	C	*S. putrefaciens*	*Enterococcus casseliflavus*, *Bacteroides uniformis*	81	F	NS	Bile	Dyslipidaemia, DM, sphincter incontinence, senile dementia	Abdominal pain; faecal vomiting; constipation; sepsis from peripheric insertion central catheter	Ciprofloxacin+fluconazole	Favourable
25	12/08/14	C	*S. algae*	N	43	M	**Y**	SST (wound)	Polytrauma after impact with a zodiac’s helix (thorax, abdomen, pelvis and right arm)	Wound infection in the right arm	Meropenem+linezolid (empirical); changed to ciprofloxacin after antibiogram	Favourable
26	25/02/15	C	*S. algae*	*K. oxytoca*, *Finegoldia magna*	36	M	NS	SST (surgical wound)	Smoker, dyslipidaemia	Infection and necrosis signs in the palmar and radial regions of the right-hand thumb after car outrage	Amoxicillin/clavulanic acid at the Emergency Unit; after surgery – ciprofloxacin+trimethoprim/sulfamethoxazole	Favourable
27	11/08/15	C	*S. algae*	*Staphylococcus aureus*	67	M	NS	SST (ulcer)	Smoker, AHT, IC	Dry necrosis in feet, leg and thigh; superinfected pretibial vascular ulcer	Meropenem	Supracondylar amputation; otherwise favourable
28	17/09/15	C	*S. algae*	*Staphylococcus aureus*, *B. fragilis*, *Clostridium perfringens*	23	M	**Y (patera)**	SST (wound)	–	Left-leg ulcers	NS	NS
29	21/09/15	C	*S. algae*	*E. coli*, *M. morganii*	15	M	**Y (patera)**	SST (ulcer)	Slight dehydration, rhabdomyolysis, patera foot; past infection by cytomegalovirus and hepatitis B; papulo-pustular lesions in the inferior limbs	Burn in the lower right limb with phlyctenas; fever, tumefaction	Imipenem+daptomycin+ clindamycin; changed to levofloxacin+ceftriaxone after antibiogram	Favourable
30	18/05/16	NSC	*S. algae*	N	60	F	NS	SST (surgical wound)	Obesity, smoker, diverticular disease, Arnold–Chiari malformation, waiting for abdominal surgery	Desloughing, malodorous wound with necrotic signs; no fever, stable.	Piperacillin/tazobactam	Favourable
31	07/07/16	C	*S. algae*	*Staphylococcus aureus*	68	M	NS	SST (abscess)	DM, arrhythmia; diabetic foot; left leg amputated (infra-condylar)	Pain, cyanosis and necrosis in right foot	Amoxicillin/clavulanic acid+metronidazole	Infra-condylar amputation of right leg; otherwise favourable

AHT, Arterial hypertension; C, community; DM, diabetes mellitus; DVT, deep venous thrombosis; F, female; IC, ischaemic cardiomyopathy; M, male; N, no; NS, not stated; NSC, nosocomial; SST, skin and soft tissue; XDR, extensively drug resistant; Y, Yes.

*The patient was not exposed to seawater directly, but to sand from a local park.

**Fig. 1.**
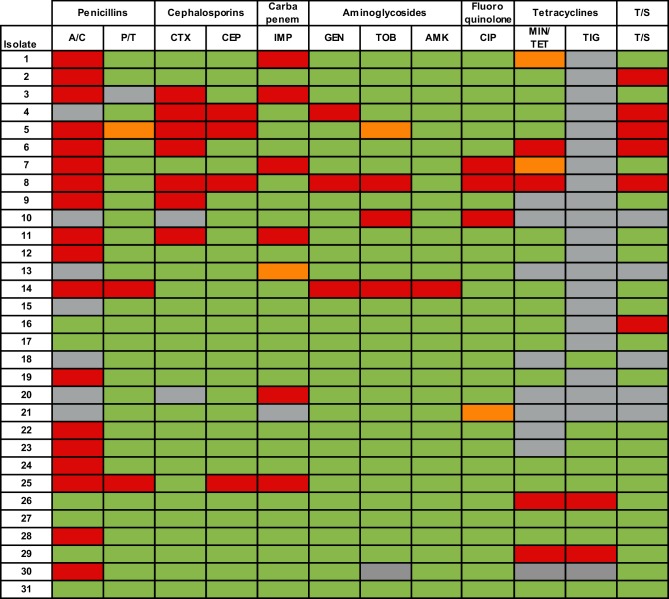
Heatmap showing the antibiotic-susceptibility profile of *S*. *algae* and *S. putrefaciens* isolates from Gran Canaria, Spain. Strain numbers refer to case numbers in Table 1. A/C, amoxicillin/clavulanic acid; P/T, piperacillin/tazobactam; CTX, cefotaxime; CEP, cefepime; IMP, imipenem; GEN, gentamicin; TOB, tobramycin; AMK, amikacin; CIP, ciprofloxacin; MIN/TET, minocycline/tetracycline; TIG, tigecycline; T/S, trimethoprim/sulfamethoxazole. Colour code: red, resistant; orange, intermediate; green, susceptible; grey, not tested.

Antimicrobial-susceptibility testing revealed several multidrug-resistant isolates (e.g. strains 5, 7, 8, 14; Fig. 1). Remarkably, uncommon for *S. algae* and *S. putrefaciens*, a number of isolates were highly resistant to aminoglycosides, cephalosporins and ciprofloxacin. In addition, sensitivity to amoxicillin/clavulanic acid, tested for in 24 strains, unravelled 18 resistant isolates. A higher number of susceptible isolates was observed for piperacillin/tazobactam. A combination of tetracyclines, β-lactams, cephalosporins and/or quinolones was required for the successful treatment of most infections in this case series.

### Literature review

Here, we review 34 reports of *Shewanella* infection from the literature between 2013 and 2016 in the literature [5–35]. In 2013, two extensive retrospective studies from Liu *et al*. [36] and Vignier *et al*. [37], including a literature review, were reported. Therefore, the selected timeframe of our review aims to provide an update of the new cases reported since then. The reader is referred to the paper by Srinivas *et al*. [38] for a prospective study (2010–2014) of *Shewanella* spp. skin and soft-tissue infections in Kerala, India.

The mean age of patients was 52.1 years (range 0–92 years). Twenty-four patients (70.6 %) were men. Most infections occurred in countries with warm climates, which included the Mediterranean region, South-Eastern Asia and the Caribbean. In most cases, the patients presented an underlying condition, such as cardiovascular disease (12 cases), diabetes (9 cases), hepatobiliary disease (6 cases), renal disease (5 cases), cancer (5 cases) and respiratory disease (2 cases). Less than half of the patients (44.1 %) reported recent contact with seawater or consumption of seafood. Clinical presentations were mostly skin and soft-tissue infections (12 cases, 30 %), gastrointestinal infections (10 cases, 25 %), bacteraemia (6 cases, 15 %) and respiratory infections (4 cases, 10 %). Two cases of immunocompromised individuals were reported. Only for five patients was there no basal pathology or underlying condition. Twenty-two infections (56.4 %) were attributed to *S. algae*, whereas 35.9 % were associated with *S. putrefaciens*. Of note, two cases of infection were by *Shewanella haliotis* [31, 39] and one case by *Shewanella xiamenensis*, isolated from rectal swabs of a 1-year-old child [12]. *S. xiamenensis* was not the causative agent of the infection, but its intestinal carriage mimicked the presence of a pathogenic *Klebsiella pneumoniae* strain that caused a previous infection in the patient. Nine isolates (23.1 %) were associated with a polymicrobial infection. A summary of the epidemiological data of the patients, clinical manifestations and antibiotic-susceptibility profiles of the isolates is presented in Tables S1–S3, available with the online version of this article.

## Discussion

Infections caused by *Shewanella* spp. are being continuously reported, with the causative agent being mainly *S. algae*. Of note, isolates from 2000 to 2007 in our case series were identified as *S. putrefaciens*, potentially due to experimental limitations [3]. In contrast, 81 % of cases retrieved from 2008 were identified as *S. algae*. State-of-the-art methods can discriminate between *S. algae* and *S. putrefaciens* on the basis of their biochemical and phenotypic characteristics [1]. Similar tests have been reported, though, to be inconclusive for discrimination of *S. algae* from other emerging pathogenic *Shewanella*, thereby requiring molecular techniques that include 16S rRNA and/or *gyrB* DNA sequencing for accurate species identification [39, 40]. Byun *et al*. reported the inability to discriminate between *S. algae* and *S. haliotis* on the basis of biochemical and phenotypic assays [39]. Even 16S rRNA sequencing, not routinely conducted in the clinical microbiology laboratory, can be insufficient to determine the identity of an isolate at the species level (E. Fernández-Fernández *et al*., unpublished results). Characteristics that can contribute to the virulence of *S. algae* are the presence of haemolysin genes, the ability to adhere to human epithelial cells, biofilm formation and exotoxin production, including tetrodotoxin [11], which might be associated with food intoxications [41].

A total of 14 of 31 *Shewanella* isolates in the case series were part of polymicrobial infections in combination with other opportunistic Gram-positive and Gram-negative pathogens (e.g. *Escherichia coli*, *Staphylococcus aureus*, *Klebsiella oxytoca*). In patient 14, *Aeromonas salmonicida* was co-cultured with *S. algae. A. salmonicida*, a bacterium of marine origin and a common fish pathogen, cannot grow at temperatures above 35 °C [42] and, consequently, its survival within the human body is compromised. Recently, infections by *A. salmonicida,* identified with the mini API and Vitek-2, have been reported from India [43, 44]. We report here the recovery of *A. salmonicida* from a skin lesion from a patient with poor health condition who had been at sea for several days. The skin has a temperature of around 30 °C, which, in combination with the infection history, may have contributed to a successful colonization.

In general, *Shewanella* infections are more frequent in males than females, although it is not yet known if this is a consequence of genetic or socio-cultural factors with a higher exposure rate [1]. Also in our case study, 75 % of the patients were men, with nine patients being irregular sub-Saharan immigrants. Thus, there are two clearly defined groups of patients. On the one hand, young otherwise healthy immigrants who acquired the infection after a prolonged stay at sea. On the other hand, local people with multiple underlying conditions, who developed an infection without documented contact with seawater and seafood consumption.

Septic shock, dehydration, rhabdomyolysis and multiple skin lesions were common conditions among the irregular travellers after their ocean journey on small, rudimentary fishing boats, termed pateras. In fact, a condition termed ‘patera foot’ has been coined to describe *Shewanella* skin and soft-tissue infections in the lower limbs of these patients, likely from a pre-existing skin lesion that comes into contact with contaminated water. Those patients were generally young, with a good health status before travelling [45]. Infections, caused by *S. algae* in 66.7 % of the patients, were very aggressive, involving cellulitis, abscesses and necrosis. Surgical debridement was carried out, but the response to broad-spectrum antimicrobial chemotherapy was generally poor. Amputation of one or several fingers was necessary in most cases. Recovery was very slow, but otherwise favourable. Among local people, 14 patients presented underlying hypertension, hypercholesterolaemia, obesity or diabetes, complicated with chronic cardiovascular conditions or cancer. These risk factors have been associated with *Shewanella* spp. and other opportunistic infections [1].

*Shewanella* infections have been frequently associated with coastal areas and warm climates. Located in the sub-tropical Atlantic Ocean, the Canary Islands match such a profile, even though the mean water temperature of around 21 °C is lower than that of other ‘hotspots’ around the equator or the tropics. Indeed, seasonal *Shewanella* infection episodes related to unusually warm conditions have been reported in Denmark [46] and Australia [47]. Even if a seasonal correlation cannot be deduced, most infections in our study were diagnosed from June to November, matching the time when a peak in the number of pateras arriving at the Canary Islands, due to the good weather conditions at sea, is registered. Sea contact, even with materials placed at sea or consumption of seafood, which are reported risk factors for *Shewanella* infections, however, was not reported for most patients. The only case from a local person with documented contact with seawater was a 43-year-old man who impacted with the helix of an inflatable boat while diving. The patient suffered multiple traumatic injuries from which *S. algae* was isolated in pure culture.

Remarkably, six patients were hospitalized at the time of the infection, therefore 19.4 % of infections were potentially nosocomial. The source of the infection could be identified in only one case (patient 30). The *S. algae* isolate was obtained several days after admission, without clear signs of infection. In this afebrile patient, a surgical wound abscess had been vacuum-drained. To avoid obstructions, the vacuum-assisted closure had been washed regularly, and on some occasions surgeons made replacements of the device. Washings had been performed with hypertonic saline solution, thereby contributing to the presence of the *S. algae* isolate.

Shewanellae are susceptible to most antibiotics, including aminoglycosides, fluoroquinolones, extended-spectrum cephalosporins, β-lactamase inhibitors, carbapenems, macrolides, aztreonam and trimethoprim/sulfamethoxazole [48]. However, the emergence of resistant strains is a concern [49, 50], as highlighted for several isolates in our case series. The antibiotic susceptibility of the isolates in this case series was highly variable including a number of multidrug-resistant strains (Fig. 1). Seventeen isolates were resistant to amoxicillin/clavulanic acid. Given that clavulanic acid is an efficient inhibitor of class A β-lactamases [51], such resistance suggests the presence of class B, C and/or D β-lactamases, as reported to be present in several *Shewanella* spp. including *S. algae* [52–54]. Resistance to a combination of piperacillin/tazobactam, with a broader spectrum than amoxicillin, was observed, as well as resistance towards the third-generation cephalosporin cefotaxime and the fourth-generation cephalosporin cefepime. Resistance to aminoglycosides is uncommon in *Shewanella* spp.; however, resistance to gentamicin, tobramycin and amikacin was observed. Indeed, the amikacin-resistant isolate (14) exhibited broad-spectrum resistance to all the tested penicillins and aminoglycosides. Several strains also displayed resistance towards antibiotics typically effective towards shewanellae, including carbapenems, tetracyclines, trimethoprim/sulfamethoxazole and fluoroquinolones (Fig. 1). Also of note is the marked differences in the antimicrobial-resistance profile observed in the isolates from patients univocally exposed to seawater during the same trip (e.g. 12–14 and 18–19), indicating that these infections involved different *S. algae* strains.

*S. algae* is in fact the environmental reservoir of *qnrA* genes that confer resistance to quinolones [55, 56]. Four variants of this gene *(qnrA2-5*) have been found to be chromosomally encoded in *S. algae* [57]. Since quinolones are extensively used in aquaculture and are stable in seawater, selective evolutionary pressure transfers these genes to enterobacteria [57, 58], giving rise to the widespread problem of plasmid-mediated resistance to quinolones in Enterobacteriaceae [59, 60]. Furthermore, *S. algae* is frequently reported to be resistant to colistin [3, 61]. Resistance to colistin in *S. algae* is mediated by the expression of ethanolamine phosphotransferase (EptA), which alters the lipopolysaccharide in the outer membrane [62].

The literature review of the last 4 years gives further support to the findings from our case series, such as the abundance of patients without recent contact with aquatic environments and/or consumption of products of marine origin. Consequently, the origin of the isolates is frequently unknown and might suggest a more widespread distribution of pathogenic *Shewanella* spp. than previously thought. Water supply systems, soil or foods can be contaminated with *Shewanella* spp. A recently reported case of intestinal colonization by *S. xiamenensis* suggests temporary colonization of the human host by *Shewanella* spp. and transfer of *bla_OXA_*_-48_-like genes to *K. pneumoniae* [12].

Even though *Shewanella* spp. infections in humans are scarce, the number of reports has significantly increased over the last decade, suggesting that species within the *Shewanella* genus, in particular *S. algae*, have indeed a pathogenic potential. *Shewanella* spp., particularly in warm coastal areas, can be considered emerging pathogens.

Herein, we have reported a case series in Gran Canaria, Spain. In a significant number of cases, the patients were irregular immigrants with patera foot syndrome, developed after prolonged exposure to seawater, which often led to amputation, followed by a complex recovery process. The response to broad-spectrum antibiotics, generally, third or fourth generation cephalosporins or alternatively, aminoglycosides, piperacillin/tazobactam, ciprofloxacin or tigecycline, recommended as an empirical therapy [63] was poor. In general, a concerning antibiotic-resistance profile that includes resistance to these commonly used antibiotics has been observed. Indeed, the arising of antibiotic resistance in *Shewanella* spp. is a concern, even if a relatively low number of infections have been reported. The genetic markers that confer such resistance, which may be horizontally transferred to other pathogenic bacteria, are still poorly characterized.

An interesting finding is the ability of *Shewanella* to colonize the human body. *Shewanella* spp. are known for their broad respiratory versatility. In an anoxic microenvironment such as the intestinal tract, *Shewanella* spp. may take advantage of the abundance of terminal anaerobic electron acceptors to colonize such niches. Indeed, several publications describe *Shewanella* spp. colonization in marine animals [64–66]. The presence of shewanellae in a variety of human tissues has been reported in the context of different microbiome studies [67–69]. It is not clear, though, whether this is the result of a transient colonization or a more permanent condition. Interestingly, a recent study in patients with and without sphincter of Oddi laxity strengthens the latter hypothesis [70]. Such colonization might also explain the high proportion of cases unrelated to recent contact with seawater. Certainly, a better understanding of the microbial ecology of the genus *Shewanella* and its pathogenic potential is required.

## Supplementary Data

308 Supplementary File 1
