# Supplementary material for: Shewanella spp. infections in Gran Canaria, Spain: retrospective analysis of 31 cases and a literature review
Source: JMM Case Rep. 2017 Dec 7;4(12):e005131. doi: 10.1099/jmmcr.0.005131 (PMC5857365; doi:10.1099/jmmcr.0.005131)
Supplement: Supplementary File 1 [file jmmcr-4-5131-s001.pdf]

Table S1.

Characteristics	No. positive
**Age**	
<18	3
18-65	19
>65	12
**Sex**	
Male	24
Female	9
Not reported	1
**Country of infection**	
USA	6
Spain	5
India	5
China	2
Taiwan	2
Thailand	2
Barbados	1
Bulgaria	1
Colombia	1
France	1
Germany	1
Greece	1
Italy	1
Iran	1
Korea	1
Malaysia	1
UK	1
Croatia	1
**Total**	34

Table S2.

	**No. positive/No. tested (%)**
**Condition***	
None	5/34 (14.7)
Immunocompromised	3/34 (8.8)
Underlying condition, of which:	22/34 (64.7)
Diabetes	9/22 (40.9)
Hepatobiliary disease	6/22 (27.2)
Renal disease	5/22 (22.7)
Cancer	5/22 (22.7)
Respiratory disease	2/22 (9.1)
Cardiovascular disease	12/22 (54.5)
**Water exposure**	
Yes	15/34 (44.1)
No	12/34 (35.3)
Not reported	7/34 (20.6)
**Type of infection****	
Skin and soft tissue	12/40 (30.0)
Bacteremia	6/40 (15.0)
Gastrointestinal	10/40 (25.0)
Respiratory	4/40 (10.0)
Other	6/40 (15.0)

* Most patients presented more than one underlying condition. For some patients, the underlying condition was not reported.
** Some patients presented more than one type of infection.

Table S3.

	No. positive/No. tested (%)
Shewanella* isolates	
S. algae	22/39 (56.4)
S. putrefaciens	14/39(35.9)
S. haliotis	2/39 (5.1)
*S. xiamenensis***	1/39 (2.6)
**Polymicrobial infections*****	9/39 (23.1)

**Antibiotic susceptibility**	
**Aminoglycosides**	
Gentamicin	18/20 (90)
Tobramycin	7/9 (77.8)
Amikacin	13/15 (86.7)
Netilmycin	1/1 (100)
**Beta lactams**	
**Penicillins**	
Ampicillin	0/6 (0)
Piperacillin	4/6 (66.7)
Amoxicillin	1/2 (50)
Carbenicillin	1/1 (100)
Ticarcillin	1/1 (100)
**Monobactams**	
Aztreonam	4/5 (80)
**Cephalosphorins**	
First generation:	
Cefalotin	0/4 (0)
Cefazolin	1/4 (25)
Cefalexin	0/1 (0)
Second generation:	
Cefuroxime	1/1 (100)
Third generation:	
Ceftriaxone	9/11 (81.8)
Cefotaxime	9/11 (81.8)
Ceftazidime	22/26 (84.6)
Cefoperazone	1/1 (100)
Cefixime	1/1 (100)
Ceftizoxime	0/1 (0)
Cefmetazole	0/1 (0)
Fourth generation:	
Cefepime	10/12 (83.3)
**Carbapenemes**	
Meropenem	17/20 (85)
Imipenem	13/17 (76.5)
Doripenem	1/1 (100)
Ertapenem	1/2 (50)
**Beta-lactams associated to beta-lactamase inhibitors**	

Ampicillin/sulbactam	1/2 (50)
Ampicillin/clavulanic acid	0/1 (0)
Piperacillin/tazobactam	13/16 (81.2)
Amoxicillin/clavulanic acid	0/4 (0)
Ticarcillin/clavulanic acid	3/6 (50)
Cefoperazone/sulbactam	1/1 (100)
**Quinolones**	
First generation:	
Nalidixic acid	2/2 (100)
Second generation:	
Ofloxacin	2/2 (100)
Norfloxacin	2/2 (100)
Ciprofloxacin	17/22 (77.3)
Pefloxacin	0/1 (0)
Third generation:	
Gatifloxacin	2/2 (100)
Levofloxacin	11/12 (91.7)
Fourth generation:	
Moxifloxacin	1/1 (100)
**Macrolides**	
Azithromycin	1/1 (100)
Moxifloxacin	2/2 (100)
Erythromycin	1/1 (100)
**Sulfonamides**	
Trimethoprim	1/1 (100)
Trimethoprim/sulfamethoxazole	12/15 (80)
**Tetracyclines**	
Tetracycline	1/3 (33.3)
Tigecycline	3/3 (100)
Doxycycline	0/1 (0)
Minocycline	5/5 (100)
**Glycopeptides**	
Vancomycin	0/1 (0)
**Polypeptides**	
Colistin	3/5 (60)
Polymyxin B	0/1 (0)
**Others**	
Chloramphenicol	4/4 (100)
Rifampicin	1/1 (100)
Nitrofurantoin	3/3 (100)

*More than one strain was recovered from some patients.
**Not properly an infectious agent, see text for details.
***In some patients, the same isolate was retrieved for more than one infection.
